# Melatonin prevents deterioration in quality by preserving epigenetic modifications of porcine oocytes after prolonged culture

**DOI:** 10.18632/aging.101680

**Published:** 2018-12-10

**Authors:** Junyu Nie, Peng Xiao, Xuefang Wang, Xiaogan Yang, Huiyan Xu, Kehuan Lu, Shengsheng Lu, Xingwei Liang

**Affiliations:** 1 State Key Laboratory for Conservation and Utilization of Subtropical Agro-Bioresources, Guangxi University, Nanning, Guangxi 530004, PR China; 2 College of Animal Science and Technology, Guangxi University, Nanning, Guangxi 530004, PR China

**Keywords:** oocyte, prolonged-culture, DNA methylation, histone methylation, melatonin

## Abstract

Prolonged culture of metaphase II oocytes is an *in vitro* aging process that compromises oocyte quality. We tested whether melatonin preserves epigenetic modifications in oocytes after prolonged culture. The porcine oocytes were maturated *in vitro* for 44 h, and then metaphase II oocytes were continuously cultured in medium supplemented with or without melatonin for 24 h. We found that the parthenogenetic blastocyst formation rate of prolonged-culture oocytes was lower than in fresh oocytes. We further observed that methylation at H3K4me2 and H3K27me2 of oocytes enhanced after prolonged culture. However, 5mc fluorescence intensity was lower in prolonged-culture oocytes than in fresh oocytes. Moreover, the promoter of the imprinted gene *NNAT* exhibited a higher level of DNA methylation in prolonged-culture oocytes than in fresh oocytes, which was associated with a reduced expression level and glucose uptake capability. Conversely, melatonin improved blastocyst formation rate and preserved histone and DNA methylation modifications, as well as *NNAT* function in the oocytes after prolonged culture. Notably, DNA methyltransferase inhibitor 5-aza significantly attenuated the protective role of melatonin on genomic DNA methylation. In summary, our results revealed that epigenetic modifications are disrupted in oocytes after prolonged culture, but the changes are reversed by melatonin.

## Introduction

Factors within oocytes are essential for successful reproduction. However, the oocyte is vulnerable to the adverse endogenous and/or exogenous environment, leading to impaired quality and compromised development. For example, in postovulatory oocyte aging, a process where fertilization does not occur within the ideal window period of post ovulation, the unfertilized metaphase II (MII) oocyte will undergo a time-dependent deterioration *in vivo* or *in vitro* [1,2], thereby leading to declined fertilization capability [3,4] and compromised early embryo development [5]. Moreover, oocyte aging also has detrimental effects on fetus development during mid-term gestation [6] and fitness of the offspring [7,8], demonstrating that postovulatory oocyte aging has profound impacts on offspring health. Therefore, it is important to fertilize the oocyte when it is fresh or to protect oocytes from deterioration when delayed fertilization is performed.

In view of the long-term effects associated with oocyte aging that originates from defects within oocytes, various attempts have been carried out to prevent oocyte deterioration during postovulatory aging, including the modification of the components of culture medium or supplementation with specific compounds. For instance, melatonin (N-acetyl-5-methoxytryptamine), a major product secreted by the pineal gland, which participates in the entrainment of the circadian rhythms and seasonal reproduction in animals [9], has been successfully applied to protect oocytes from aging *in vitro* [3,4,10,11]. Previous reports show that oxidative stress is induced during postovulatory aging [12], which leads to impaired functions in oocytes including disrupted spindle assembly, chromosome alignment, actin polymerization, and mitochondrial integrity [13,14]. Therefore, fertilization capability and the competence of early embryo development are reduced [4,10,13]. Being a free radical scavenger and potent antioxidant [15], melatonin vastly reduces reactive oxygen species (ROS) level and attenuates the defects of postovulatory aging oocytes. However, the molecular process by which melatonin delays oocyte postovulatory aging still needs to be further elucidated.

Epigenetic modifications are mechanisms that regulate gene expression independently of the DNA sequence. Genomic DNA methylation and histone modification are widely-studied epigenetic mechanisms. DNA methylation occurs on the 5-position cytosine, which suppresses gene expression, while demethylation activates gene expression. DNA methylation patterns are established during oogenesis to maintain subsequent development and offspring fitness [16]. Methylation not only takes place at the genomic DNA level but also at the loci of the histone lysine. In contrast to DNA methylation, histone methylation can switch gene expression on or off depending on which lysine loci are methylated. In general, methylation at H3K4 is associated with gene activation, but methylation at H3K27 is associated with gene repression. Both the modification of DNA and histones by methylation play important roles in oogenesis and early embryogenesis [17,18]. Defects of epigenetic modifications may contribute to compromised oocyte quality during postovulatory aging [19,20]. We and others have observed defective epigenetic modifications in postovulatory aging mouse and porcine oocytes [5,21–23], whether these epigenetic abnormalities can be reversed needs to be further explored [19].

In the present study, we determined whether melatonin preserves epigenetic modifications in oocytes during *in vitro* aging. We observed that histone methylation, global genomic DNA methylation, and DNA methylation of individual gene were disrupted in porcine MII oocytes after prolonged culture. Moreover, gene expression and gene function was further altered and associated with hypermethylation level at the *NNAT* promoter. Conversely, melatonin could fully or partially reverse the altered epigenetic modification parameters in prolonged-culture oocytes. We report that melatonin could preserve epigenetic in oocytes after prolonged culture.

## RESULTS

### Melatonin attenuates the deterioration in the quality of prolonged-culture oocytes

**Figure 1:**
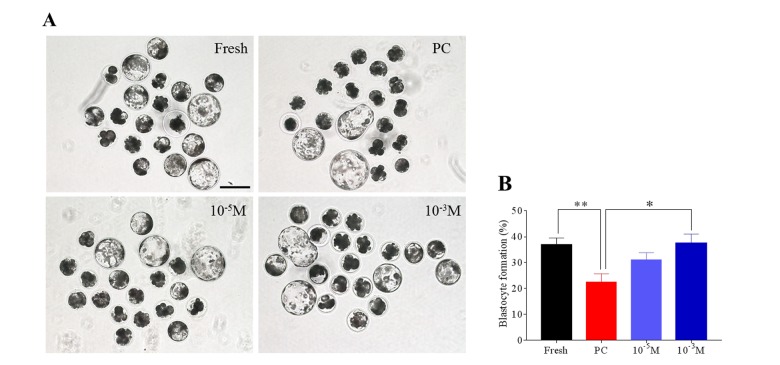
**Melatonin enhances the blastocyst formation rate of prolonged-culture oocytes.** Porcine oocytes matured *in vitro* were continuously cultured in medium supplemented with or without melatonin (10^−3^ or 10^−5^ M) for 24 h. Oocytes were pathogenetically activated and then cultured for 7 days to examine the blastocyst formation rate. Views of blastocysts (**A**) and blastocyst formation rate (**B**) of fresh, prolonged-culture, and prolonged-culture + Mel oocytes. The data are presented as the mean ± SEM of at least three independent experiments. Bar=200 μm; PC, prolonged-culture; Mel, melatonin; *P<0.05, **P<0.01.

A high-quality oocyte is essential for early development, therefore we tried to determine the protective role of melatonin on the quality of prolonged-culture oocytes via evaluating the blastocyst formation rate. To do this, fresh, prolonged culture (prolonged-culture) and prolonged culture + Mel (Melatonin, 10^−3^ or 10^−5^ M) oocytes were parthenogenetically activated and then cultured *in vitro* to check blastocyst formation. As shown in Figure 1A and B, the blastocyst formation rate of the prolonged-culture oocytes was lower than the fresh oocytes (Fresh, 37.0 ± 2.6%; prolonged culture, 22.5 ± 3.2%; *P*<0.01), indicating oocyte quality is impaired after prolonged culture. Conversely, melatonin supplement at 10^−5^ M and 10^−3^ M concentration increased blastocyst formation rate to 31.0 ± 2.9% and 37.4 ± 3.6%, respectively. Collectively, our data showed that melatonin enhances blastocyst formation of prolonged-culture oocytes, suggesting that melatonin prevents deterioration. A melatonin concentration of 10^−3^ M significantly improved blastocyst formation rate of the prolonged-culture oocytes (prolonged culture, 22.5 ± 3.2%; prolonged culture + Mel, 37.4 ± 3.6%; *P*<0.05), therefore this concentration was used in subsequent experiments.

### Melatonin preserves the histone methylation of prolonged-culture oocytes

**Figure 2:**
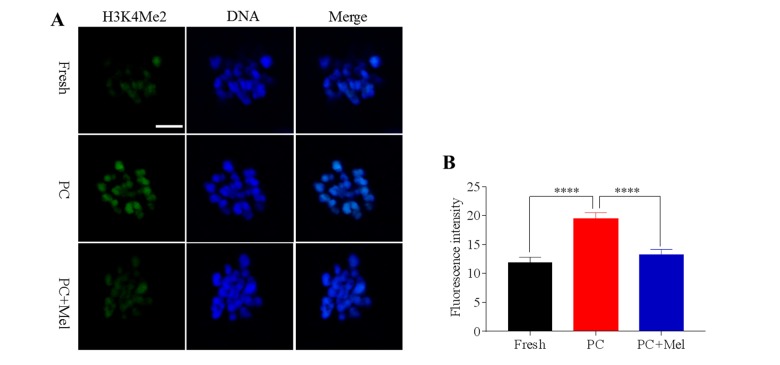
**The effect of melatonin on histone methylation at H3K4me2 of prolonged-culture oocytes.** Porcine oocytes matured *in vitro* were continuously cultured in medium supplemented with or without 10^−3^ M melatonin for 24 h. (**A**) Representative images of fresh, prolonged-culture and prolonged-culture + Mel oocytes stained with H3K4me2 antibody. (**B**) Quantitative analysis of H3K4me2 fluorescence intensity. The data are presented as mean ± SEM of at least three independent experiments. PC, prolonged-culture; Mel, melatonin; Scale bar=100 μm. Data are expressed as the mean ± SEM, ****P<0.0001.

**Figure 3:**
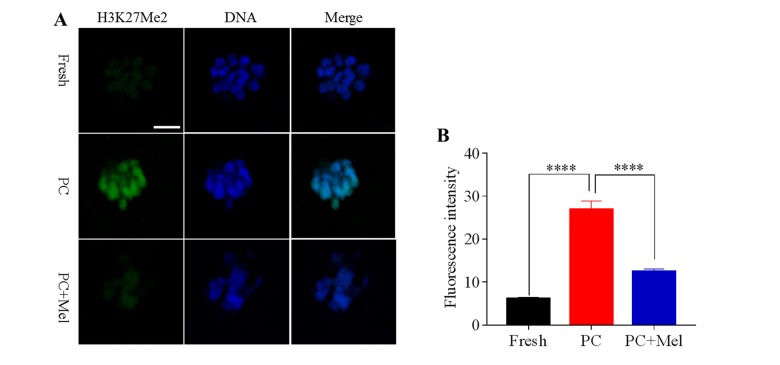
**Effect of melatonin on histone methylation at H3K27me2 of prolonged-culture oocytes.** Porcine oocytes matured *in vitro* were continuously cultured in medium supplemented with or without 10^−3^ M melatonin for 24 h. (**A**) Representative images of fresh, prolonged-culture and prolonged-culture + Mel oocytes stained with H3K27me2 antibody. (**B**) Quantitative analysis of H3K27me2 ﬂuorescence intensity. PC, prolonged-culture; Mel, melatonin, Scale bar=100 μm. The data are expressed as the mean ± SEM of at least three independent experiments, **** *P*<0.0001.

To test whether histone methylation in oocytes is influenced by prolonged culture and melatonin, oocytes were stained with antibody H3K4me2 and H3K27me2. As shown in Figure 2A and B, we found that the fluorescence intensity of H3K4me2 was higher in the prolonged-culture oocytes compared to the fresh oocytes (Fresh, 11.9 ± 0.9; prolonged culture 19.4 ± 1.1; *P*<0.0001), and the melatonin supplement reduced the fluorescence intensity of H3K4me2 in the prolonged-culture oocytes (prolonged culture, 19.4 ± 1.1; prolonged culture + Mel, 13.3 ± 0.9; *P*<0.0001). Similarly, as shown in Figure 3A and B, fluorescence intensity of H3K27me2 was higher in the prolonged-culture oocytes compared to the fresh oocytes (Fresh, 6.1 ± 0.3; prolonged culture, 26.9 ± 2.0, *P*<0.0001), but melatonin reduced the fluorescence intensity of H3K27me2 in the prolonged-culture oocytes (prolonged culture, 26.9 ± 2.0; prolonged culture + Mel, 12.4 ± 0.6; *P*<0.0001). Taken together, the above results suggest that histone methylation of the oocytes is disrupted by prolonged culture, however, melatonin protects against the defect.

### Melatonin attenuates global genomic DNA methylation defects in prolonged-culture oocytes

**Figure 4:**
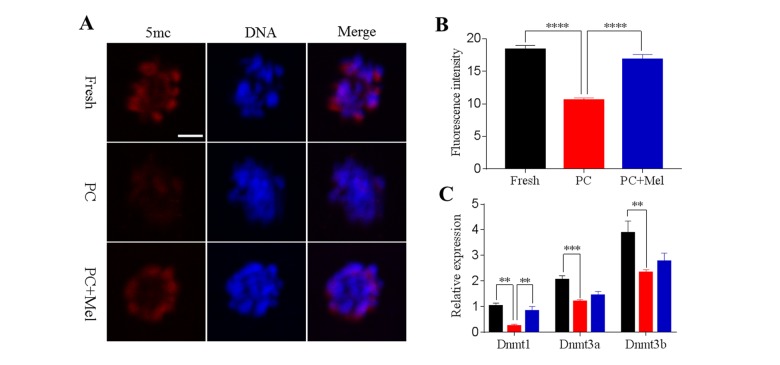
**Effects of melatonin on global genomic DNA methylation and DNMT expression in prolonged-culture oocytes.** Porcine oocytes matured *in vitro* were continuously cultured in medium supplemented with or without 10^−3^ M melatonin for 24 h. (**A**) Representative images of fresh, prolonged-culture, and prolonged-culture + Mel oocytes stained with 5mc antibody. (**B**) Quantitative analysis of 5mc ﬂuorescence intensity. (**C**) Expression of *DNMT1*, *DNMT3A*, and *DNMT3B* in fresh, PC, and PC + Mel oocytes. The data are presented as the mean ± SEM of at least three independent experiments. Scale bar=100 μm; PC, prolonged-culture; Mel, melatonin; **P<0.01*,* *** *P*<0.001, **** *P*<0.0001.

DNA methylation is the most-studied epigenetic modification, which regulates gene expression independently of the gene sequence. We next tested whether DNA methylation in oocytes is influenced by prolonged culture and exposure to melatonin. The fresh, prolonged culture, and prolonged culture + Mel oocytes were stained with antibody 5mc. As shown in Figure 4A and B, the fluorescence intensity of 5mc in the prolonged-culture oocytes was significantly reduced compared to the fresh oocytes (Fresh, 18.4 ± 0.6; prolonged culture, 10.5 ± 0.4; *P*<0.0001). Interestingly, melatonin significantly increased the fluorescence intensity of 5mc in the prolonged-culture oocytes (prolonged-culture, 10.5 ± 0.4; prolonged-culture + Mel, 16.9 ± 0.7; *P*<0.0001). Because DNA methylation is mediated by DNA methyltransferase (DNMTs), we next investigated DNMT expression. As shown in Figure 4C, the expression level of *DNMT1*, *DNMT3A*, and *DNMT3B* in the prolonged-culture oocytes were significantly reduced compared to the fresh oocytes (1.0 ± 0.1, 0.2 ± 0.1, *P*<0.01; 2.1 ± 0.1, 1.2 ± 0.1, *P*<0.001; and 3.9 ± 0.5, 2.3 ± 0.1, *P*<0.01; for *DNMT1*, *DNMT3A*, and *DNMT3B*, respectively). Notably, melatonin was able to significantly increase the expression level of *DNMT1* compared to the prolonged-culture oocytes (prolonged culture, 0.2 ± 0.1; prolonged culture + Mel, 0.8 ± 0.2; *P*<0.01), and *DNMT3A* and *DNMT3B* expression was also slightly increased (prolonged culture, 1.2 ± 0.1, 2.3 ± 0.1, prolonged culture + Mel, 1.4 ± 0.2, 2.8 ± 0.3 for *DNMT3A* and *DNMT3B,* respectively), suggesting it is possible that melatonin affects DNA methylation by regulating DNMT expression.

### Melatonin preserves DNA methylation, expression and function of *NNAT* in prolonged-culture oocytes

**Figure 5:**
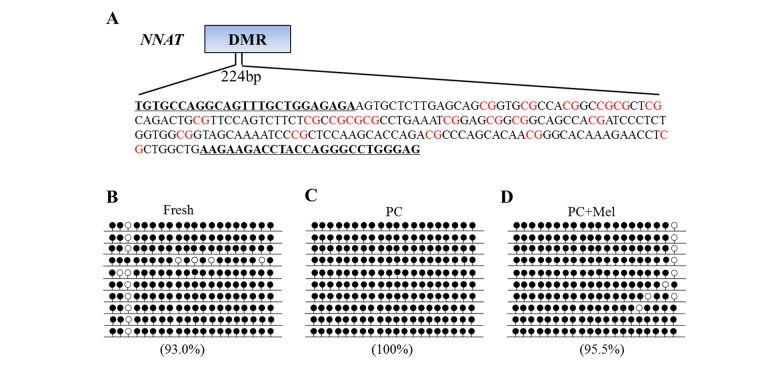
**The effect of melatonin on DNA methylation on the *NNAT* promoter of prolonged-culture oocytes.** Porcine oocytes matured *in vitro* were continuously cultured in medium supplemented with or without 10^−3^ M melatonin for 24 h. (**A**) Primer and CpG loci examined at the *NNAT* differentially methylated region (DMR) are shown. DNA methylation was examined by bisulfite sequencing in fresh (**B**), prolonged-culture (**C**) and prolonged-culture + Mel oocytes (**D**). Each line presents an individually cloned allele, with an open circle for a non-methylated CpG site and filled circles for methylated CpG. Ten clones successfully sequenced for each group are presented. Methylation level (%) = (Methylated CpG/Total CpG) × 100. PC, prolonged-culture; Mel, melatonin.

A previous study has shown that promoter DNA methylation and the function of *NNAT* are disrupted in prolonged-culture porcine oocytes [5], therefore, we used *NNAT* as an indicator to examine whether melatonin could reverse these defects (Figure 5A). To examine DNA methylation level in fresh, prolonged culture, and prolonged culture + Mel oocytes we applied bisulfite sequencing. As shown in Figure 5B-D, the DNA methylation level of the *NNAT* promoter was 93% in fresh oocytes but reached 100% in the prolonged culture oocytes. Notably, DNA methylation at the *NNAT* promoter was reduced to 95.5% in the prolonged culture + Mel oocytes. Our results show that melatonin could attenuate a DNA methylation defect at a specific promoter.

**Figure 6:**
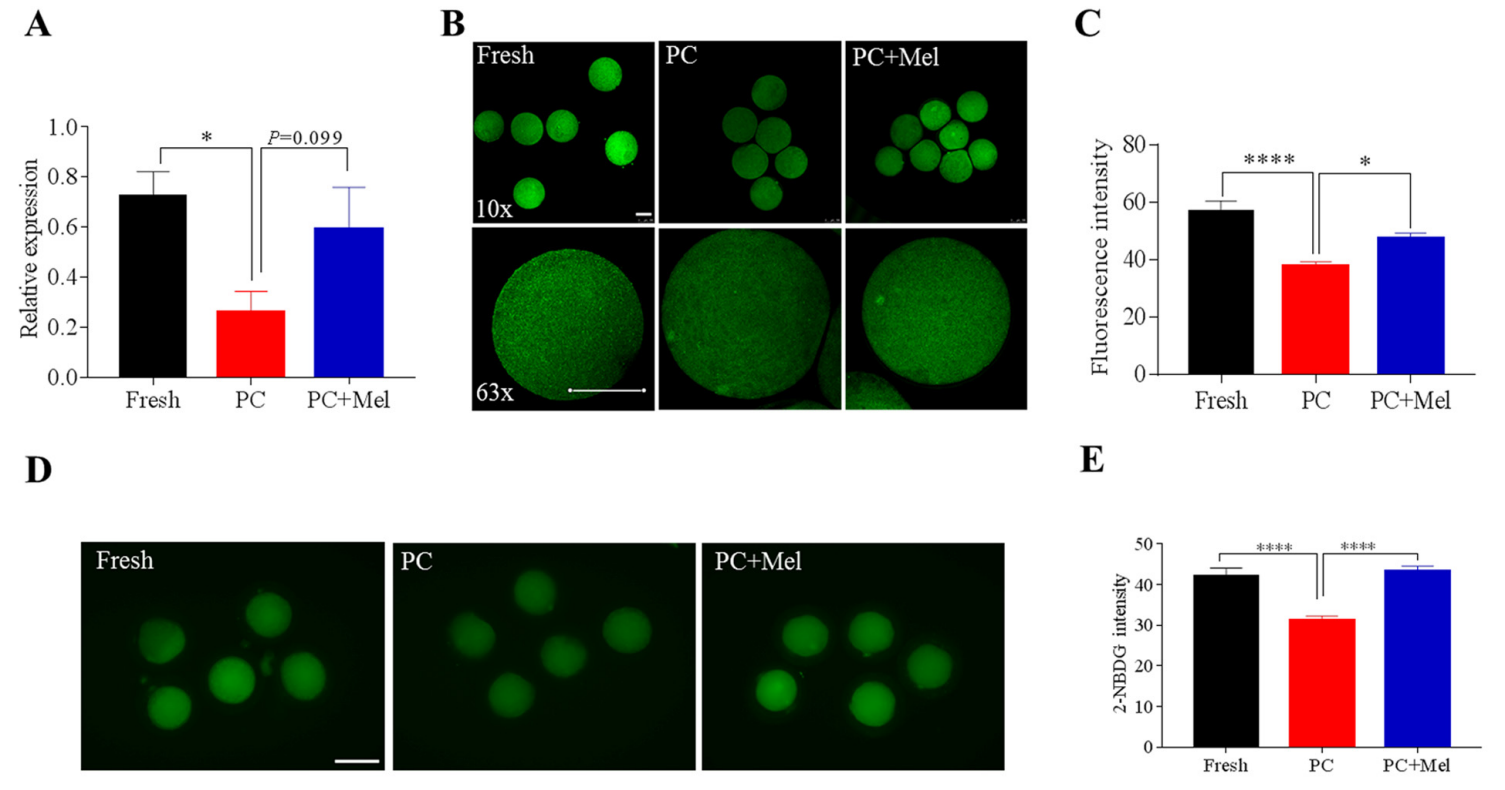
**The effect of melatonin on *NNAT* expression and the glucose uptake of prolonged-culture oocytes.** Porcine oocytes matured *in vitro* were continuously cultured in medium supplemented with or without 10^−3^ M melatonin for 24 h. (**A**) *NNAT* mRNA levels in the fresh, prolonged-culture, and prolonged-culture + Mel oocytes. (**B**) Representative images of fresh, prolonged-culture and prolonged-culture + Mel oocytes stained with NNAT antibody. (**C**) Quantification of the NNAT protein level. (**D**) Representative images of fresh, prolonged-culture and prolonged-culture + Mel oocytes stained with 2-NBDG. (**E**) Quantification of the 2-NBDG level. PC, prolonged-culture; Mel, melatonin; Scale bar=100 μm. The data are presented as the mean ± SEM of at least three independent experiments. *P<0.05, **** *P*<0.0001.

Melatonin could preserve the DNA methylation of the *NNAT* promoter in prolonged culture oocytes, therefore, we further studied how melatonin impacts *NNAT* expression and its function. We first determined the *NNAT* expression level in fresh, prolonged culture, and the prolonged culture + Mel oocytes. As shown in Figure 6A, the mRNA level of *NNAT* in the prolonged culture oocytes was significantly lower than the fresh oocytes (Fresh, 0.7 ± 0.1; prolonged culture, 0.3 ± 0.1; *P*<0.05), and slightly increased after treatment with melatonin (prolonged culture, 0.3 ± 0.1; prolonged culture + Mel, 0.6 ± 0.2; *P*=0.099). We further stained the oocytes with NNAT antibody to examine protein level. Similar to mRNA expression, fluorescence intensity of NNAT was reduced in the prolonged culture oocytes (Fresh, 56.9 ± 3.6; prolonged culture, 37.6 ± 1.6; *P*<0.0001) and increased in the prolonged culture + Mel oocyte (prolonged culture, 37.6 ± 1.6; prolonged culture + Mel, 47.1 ± 2.1; *P*<0.05), further confirming that melatonin improved *NNAT* expression (Figure 6B and C). As previous studies revealed that downregulation of NNAT inhibited glucose uptake in prolonged-culture porcine oocytes, we next examined the oocytes capability to uptake glucose after treatment with melatonin. To accomplish this, fresh, prolonged culture, and prolonged culture + Mel oocytes were incubated with 2-NBDG. As shown in Figure 6D and E, the glucose uptake level in the prolonged culture oocytes is lower than that of the fresh oocytes (Fresh, 42.2 ± 1.9; prolonged culture, 31.3 ± 1.0; *P*<0.0001), but melatonin significantly enhanced glucose uptake level (prolonged culture, 31.3 ± 1.0; prolonged culture+ Mel, 43.2 ± 1.3; *P*<0.0001). Collectively, our data indicate that melatonin preserves DNA methylation and gene function of prolonged-cultured oocytes.

### DNMT inhibitor 5-aza attenuates the protective role of melatonin on genomic 5mc in prolonged-culture oocytes

**Figure 7:**
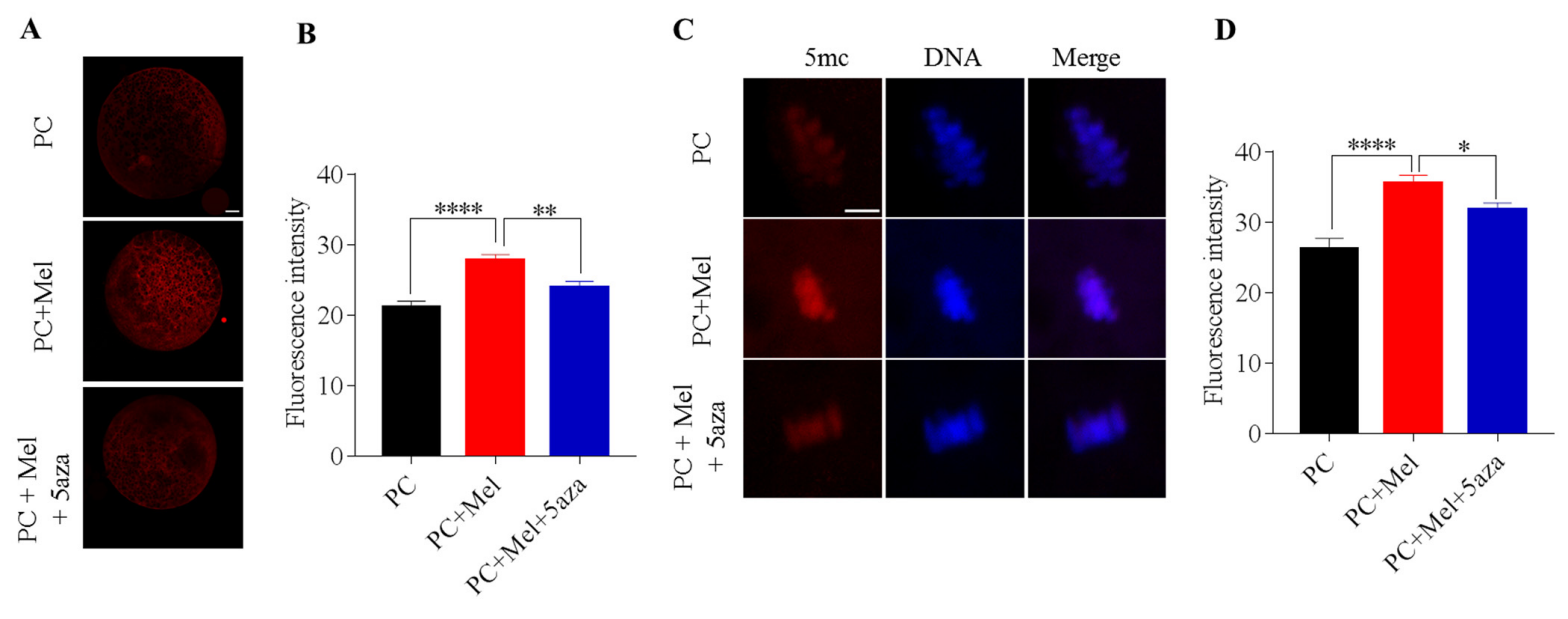
**DNA methyltransferase (DNMT) inhibitor 5-aza attenuated the protective role of melatonin on genomic DNA methylation in prolonged-culture oocytes.** Porcine oocytes matured *in vitro* were continuously cultured for 24 h or in medium supplemented with 10^−3^ M melatonin or 10^−3^ M melatonin plus 1 μM 5-aza for 24 h. (**A**) Representative images of oocytes stained with DNMT1 antibody; (**B**) Quantification of DNMT1 fluorescence intensity in the prolonged-culture, prolonged-culture + Mel, and prolonged-culture + Mel + 5-aza oocytes. (**C**) Representative images of oocytes stained with 5mc antibody; (**D**) Quantification of 5mc fluorescence intensity in the prolonged-culture, prolonged-culture + Mel, and prolonged-culture + Mel + 5-aza oocytes. Scale bar, A=25 μm, B=5 μm; PC, prolonged-culture; Mel, melatonin; *P<0.05*,* ** *P*<0.01, **** *P*<0.0001.

Given that DNA methylation is mediated by DNMTs, we tried to explore whether melatonin affects DNA methylation via activating DNMT activity in prolonged-culture oocytes. To achieve this, DNMT inhibitor 5-aza was added to the medium during prolonged culture. If DNMT activity is inhibited, the protective role of melatonin on genomic DNA methylation will be attenuated. Expectedly, we first observed that the prolonged culture + Mel oocytes have higher DNMT1 protein level compared to that of the prolonged culture oocytes (prolonged culture + Mel, 27.8 ± 0.8; prolonged culture, 21.2 ± 0.8; *P*<0.0001), as shown in Figure 7A and B. Interestingly, 5-aza attenuated the upregulation of DNMT1 protein by melatonin (prolonged culture + Mel + 5-aza, 23.9 ± 0.9; prolonged culture + Mel, 27.8 ± 0.8; *P*<0.01). We next assayed whether 5-aza may weaken the action of melatonin to preserve genomic 5mc in prolonged-culture oocytes. We stained the prolonged culture, prolonged culture + Mel, and prolonged culture + Mel + 5-aza oocytes with 5mc antibody. Consistent with our above findings, prolonged culture + Mel oocytes have higher 5mc fluorescence intensity than the prolonged culture oocytes (prolonged culture + Mel, 35.5 ± 1.2; prolonged culture, 26.3 ± 1.4; *P*<0.0001), as shown in Figure 7C and D, but 5-aza significantly reduced the upregulation of 5mc fluorescence intensity induced by melatonin (prolonged culture + Mel + 5-aza, 31.8 ± 1.0; prolonged culture + Mel, 35.5 ± 1.2; *P*<0.05). Taken together, our results suggest that melatonin preserves genomic DNA methylation through the regulation of DNMT1 expression.

## DISCUSSION

In the current study, we observed epigenetic defects, including disruption of histone methylation and DNA methylation, at overall and local levels in porcine oocytes subjected to prolonged culture. Along with previous findings, we demonstrate that the epigenetic abnormalities may contribute to the compromised quality of prolonged-culture oocytes [5,21,23,24]. Though some compounds, such as melatonin have been found to effectively delay postovulatory oocyte aging and extend the window for optimal fertilization [21,25,26], the mechanisms behind this still need to be further explored. We revealed that melatonin preserved histone methylation and DNA methylation of prolonged-culture porcine oocytes. Notably, we observed that melatonin stabilizes the modification of DNA methylation via regulating DNMT1 activity. Our findings will help us to further understand the epigenetic defects in prolonged-culture oocytes, and whether the defects are reversible. Thus, it is valuable to preserve epigenetic modifications when delayed fertilization oocytes are applied in the *in vitro* production of animal embryos and in assisted reproductive technologies in humans [2].

Histone methylation regulates gene expression and plays a crucial role in regulating oocyte growth [27,28]. In general, H3K4 methylation is related to gene activation while H3K27 methylation is associated with gene inactivation [29]. Therefore, we selected H3K4me2 and H3K27me2 as indicators to examine the impact of melatonin on histone methylation in prolonged-culture oocytes. Our results showed that both H3K4me2 and H3K27me2 gained methylation in prolonged-culture oocytes. Along with a recent study, which reported that H3K9me3 was disrupted in postovulatory aging mouse oocytes [21], our evidence demonstrates that a defect in histone methylation may be one of the molecular mechanisms contributing to the compromised quality of prolonged-culture oocyte. Exposure to toxic substances also alters histone methylation at H3K4me2 and H3K27me3 [30,31]. The evidence implies that an abnormality in histone methylation may contribute to poor oocyte quality. Therefore, it is necessary to maintain histone methylation to prevent the deterioration of oocytes. Melatonin is widely applied in a protective role against aging in oocytes but the relative molecular processes need further study. We currently investigated the effect of melatonin on histone methylation in prolonged-culture oocytes and observed that melatonin reduced histone methylation at H3K4me2 and H3K27me2. Melatonin also could reverse the defect of histone methylation at H3K9me2 in mouse oocytes exposed to deoxynivalenol during IVM [32]. Although the underlying mechanisms still need to be further explored, it is confirmed that melatonin has a protective role on histone methylation in oocytes after prolonged culture.

DNA methylation patterns of MII oocytes are established during oogenesis and oocyte growth [33,34]. Soon after fertilization, paternal pronuclei demethylates are followed by maternal pronuclei, but an imprinted gene maintains its DNA methylation pattern during this wave of demethylation [16]. If the DNA methylation patterns in oocytes or zygotes are disrupted, the subsequent development may be adversely impacted [35]. We observed that overall genomic DNA had a reduced level of methylation and that *NNAT* exhibited levels of hypermethylation at differentially methylated regions (DMRs) in prolonged-culture oocytes. We further found that hypermethylation at *NNAT* DMR inhibited glucose uptake. These findings show that not only DNA methylation but also gene function is disrupted in prolonged-culture oocytes. When cultures were supplemented with melatonin, DNA methylation at global and local levels was restored. Because melatonin may regulate gene expression by mediating epigenetic modification, we also found that *NNAT* expression is rescued in prolonged-culture oocytes [36]. Collectively, we revealed that epigenetic defects with histone and DNA methylation modifications are reversed by melatonin in prolonged-culture oocytes. However, the possible links between histone and DNA methylation and how they adversely affect oocyte quality still need to be further explored.

DNA methylation is one of the best studied epigenetic modifications [16]. We further tried to explore how melatonin restores genomic DNA methylation in prolonged-culture oocytes. The DNA methylation pattern is regulated by DNA methyltransferase, therefore, the protective role of melatonin on the modification of DNA methylation in the prolonged-culture oocyte could be mediated by DNMTs. We found that the DNMT1 protein was downregulated in the prolonged-culture oocytes, but it was reversed by melatonin. This is concomitant with the trend of global genomic DNA 5mc among fresh, prolonged culture, and prolonged culture + Mel oocytes. DNMT1 activity is majorly inhibited by 5-aza, leading to a hypomethylation status [37]. When DNMT1 activities were inhibited by 5-aza, the protective role of melatonin on genomic DNA methylation was attenuated. Moreover, a recent study demonstrates that melatonin could greatly enhance *DNMT1* expression to promote porcine oocyte maturation *in vitro* [38], suggesting that the beneficial effects of melatonin on oocytes may be mediated by DNMTs.

Of note, we observed that global genomic DNA lost methylation but some specific loci gained methylation in prolonged-culture oocytes. It appears that both are inconsistent. This could be because only one gene was examined, we could not rule out whether some other genes also lost DNA methylation as in the global genomic DNA. This is supported by our previous observation that the imprinted gene *Snrpn* lost DNA methylation in postovulatory aging mouse oocytes [23]. Another possible interpretation is that the loss of genomic DNA methylation leads to overall hypomethylation and unstable genomic DNA. Thereby, some specific loci may gain or lose methylation. Interestingly, this phenomenon is observed in tumor tissue, global genomic DNA exhibits hypomethylation status while suppressed cancer genes are hypermethylated to promote tumor growth [39,40]. In particular, melatonin treatment inhibits breast cancer tumor growth in mice through upregulating global DNA methylation [41].

The limitation of our study is that only histone and DNA methylation were investigated. There are still some other epigenetic modifications such as histone acetylation and non-coding RNA that need to be further explored in prolonged-culture and melatonin-exposed oocytes. However, our results at least provide indications that melatonin prevents oocytes from deterioration during postovulatory aging partially by preserving epigenetic modifications.

## MATERIALS AND METHODS

All chemicals and reagents used in the present study were purchased from Sigma-Aldrich (St. Louis, MO, USA) except where otherwise stated.

### *In vitro* maturation (IVM)

Porcine ovaries were obtained from a local slaughterhouse and transported to the laboratory in 0.9% saline at approximately 30ºC, as previously described [42]. Follicular fluid from antral follicles at 3–8 mm diameter was aspirated using an 18-gauge needle connected to a 10 ml disposal syringe. Cumulus-oocyte complexes (COCs) were collected and washed multiple times with polyvinyl alcohol (PVA)-TL-HEPES. COCs were matured *in vitro* in TCM-199 (Gibco) culture medium supplemented with 3.05 mM D-glucose, 0.91 mM sodium pyruvate, 0.57 mM cysteine, 0.01 μg/ml epidermal growth factor (EGF), 0.50 μg/ml follicle-stimulating hormone (FSH), 0.50 μg/ml luteinizing hormone (LH), 10% (v/v) porcine follicular fluid, 75.00 μg/ml penicillin G, and 50.00 μg/ml streptomycin. Approximately, fifty COCs were cultured per 200 μl IVM medium covered with mineral oil. COCs were cultured in an incubator with humidified air at 38.5ºC and 5% CO_2_ for 44 h_._

### Prolonged culture and treatment

At 44 h post IVM, cumulus cells were removed from COCs in PVA-TL-HEPES containing 0.1% hyaluronidase. The oocytes with intact morphology and first polar body were selected and continuously cultured in PZM-3 medium supplemented with or without melatonin for 24 h. Melatonin was dissolved in absolute ethanol and the final concentration of ethanol in the aging medium did not exceed 0.1%. Based on previous studies and our preliminary test, 10^−5^ M and 10^−3^ M melatonin were added to the PZM-3medium.

The DNMT inhibitor 5-aza was purchased from Aladdin (Shanghai, China). The 5-aza was dissolved in DMSO and the final concentration in the medium was 1 µM [37].

### Parthenogenetic activation

The parthenogenetic activation was performed using electrical pulses (2 DC pulses of 1.2 kV/cm for 30 μs) under an ECM 2001 electro cell manipulator (BTX Inc., San Diego, CA, USA) in the activation medium (0.30 M Mannitol, 1.00 mM CaCl_2_•2H_2_0, 0.10 mM MgSO_4_, 0.50 mM HEPES plus 0.3% (w/v) BSA). Activated oocytes were immediately transferred to PZM-3 and cultured in a humidified air incubator at 38.5ºC and 5% CO_2._ Blastocyst formation was examined at 168 h after activation.

### Glucose uptake assay

A fluorescent glucose analog, 2-(N-(7-nitrobenz-2-oxa-1,3-diazol-4-yl) amino)-2-deoxyglucose (2-NBDG, Beijing Chemsynlab Pharmaceutical Co. Ltd., Beijing, China; CAS 186689-07-6), was used to evaluate glucose uptake capability. Oocytes were cultured in 200 µM 2-NBDG dissolved in PBS for 30 min at 37ºC. After washing with PBS, images were captured under a fluorescence microscope (Nikon, Japan). Fluorescence intensity was quantified by using NIH Image J software to compare glucose uptake capability.

### RNA isolation and real-time PCR

Total RNA was extracted from 50 oocytes by using an RNAprep Pure Micro kit (Tiangen, Beijing, China) according to the manufacturer’s instructions. cDNA was synthesized by using a Fast Quant RT Kit (Tiangen). The real-time PCR reaction system was prepared as follows: 10 μl Super Real Premix Plus (2×) (Tiangen), 2 μl cDNA, and 0.2 μM primers. The PCRs were performed with a Bio-Rad CFX 96 (USA) unit using the following program: 1 cycle at 95ºC for 15 min, 39 cycles at 95ºC for 10 s, 60ºC for 30 s. As previously described, eGFP was added to the oocyte sample before RNA isolation. The expression of eGFP was used for normalization and relative expression levels were determined by the 2^−∆∆Ct^ method [5]. The primers used in this study are listed in Table 1.

**Table 1:** Primer sequences used for real-time PCR and bisulfite sequencing PCR (BSP).

**Gene**	**Primer sequence 5’-3’**	**Gene Access** **no./Reference**	**Length (bp)**
Dnmt1	F: GTGGCGTTTGTGAGGTTTGT R: CATCATCGTCTGCCTCCTTC	XM_021082064.1	150
Dnmt3a	F: GAATGCCACCAAATCAGCC R: GAACTTGCCGTCTCCGAAC	XM_021085534.1	193
Dnmt3b	F: ATTTGACGGGTGACGGAGAC R: TTCGGACCGCTGGACTTT	XM_021077256.1	114
Tet1	F: TCTTCCGACCTTGTCTACC R: GCTCGTCTTCTTCCACCA	NM_001315772.1	158
*NNAT*	F: CGACAATACCAGATTCCTTC R: CTTGGTCCAGATCAGAATGT	Gao *et. al.* [5]	138
BSP-*NNAT*-out BSP-*NNAT*-in	F: ATAGTAGGTGTTTAGTGGAGAG R: ATAATCACCGAATATCTACCCTAT F: TGTGTTAGGTAGTTTGTTGGAGAGA R: CTCCCAAACCCTAATAAATCTTCTT	Gao *et. al.* [5]	748 224

### Immunofluorescent staining

Oocytes were collected and washed with PBS containing 0.1% PVA then fixed with 4% paraformaldehyde (PFA) at room temperature (RT) for 30 min. The oocytes were then permeabilized with 1% Triton-100 in PBS at RT for 8–12 h.

For 5mc staining, oocytes were blocked with 1% BSA for 1 h and treated with 2 N HCl for 15 min. They were then neutralized with 100 mM Tris-HCl (PH=8.5) for 20 min at RT. After incubation in 0.05% Tween-20 for 1 h at RT, oocytes were incubated overnight at 4ºC with 5mc antibody (Zymo Research, Irvine, CA, USA) at a dilution of 1:500. After washing with 0.05% Tween-20, oocytes were incubated with anti-mouse IgG/TRITC second antibody (ZSGB-BIO, Beijing, China) at a dilution of 1:300 for 1 h at RT.

For histone methylation and Dnmt1 staining, oocytes were blocked with 1% BSA for 1 h at RT. After washing with 0.05% Tween-20, oocytes were incubated with primary antibody H3K4me2 and H3K27me2 (Cell Signaling, Danvers, MA, USA) at a dilution of 1:500, and DNMT1 (Proteitech, Chicago, IL, USA) at a dilution of 1:200 overnight at 4ºC. Then the oocytes were washed with 0.05% Tween-20 followed by incubation with secondary antibody Alexa Fluor 488 (ZSGB-BIO, Beijing, China) for 1 h at RT.

NNAT staining was performed according to a previous description [5]. Briefly, oocytes were washed three times with PBS, fixed with 4% PFA at RT for 30 min and then placed into 50% methanol for 5 min, 100% methanol for 5 min, and 100% acetone for 5 min to remove lipid droplets and then were immediately permeabilized in 1% Triton-100 overnight at RT. After blocking in 1% BSA for 1 h at RT, the oocytes were incubated with NNAT antibody (Bioss, Beijing, China) at a dilution 1:300 overnight at 4ºC. Oocytes were washed three times with 0.05% Tween-20, stained with goat anti-rabbit Alexa Fluor 488 (ZSGB-BIO, Beijing, China) at a dilution of 1:200. Then they were washed with 0.05% Tween-20 and stained with 10 mg/ml hoechst33342 for 30 min at 37 ºC. Oocytes were washed in PBS with 0.1% PVA and mounted on glass slides. Images were captured by a confocal laser scanning microscope (LAS - Leica TCS-SP8, Wetzlar, Germany). To compare the fluorescence intensity of the same antibody, the same scan settings were used in fresh, prolonged-culture, and prolonged-culture oocytes with melatonin (prolonged-culture + Mel).

### Bisulfite sequencing (BS)

Oocyte genomic DNA was modified using the EZ DNA Methylation-Direct Kit (Zymo Research) according to the manufacturer’s instructions with minor modifications as previously described [43]. To obtain PCR products, nested PCR was carried out using the following reaction system: the first round comprised 2 μl bisulfite-converted DNA, 0.5 mM primers and 12.5 μl Zymo Taq^TM^ Premix (Zymo Research) in a total volume of 25 µl. The second round of PCR was performed with 2 μl of the first-round product as template, 0.5 mM primers, and 10 μl Zymo Taq^TM^ Premix in a total volume of 20 μl. The program for the first round was 1 cycle at 95ºC for 10 min, 39 cycles of 95ºC for 30 s, 53ºC for 30 s, 72ºC for 1 min; and 1 cycle at 72ºC for 7 min. The second round PCR was performed as 1 cycle at 95ºC for 10 min, 39 cycles of 95ºC for 30 s, 56ºC for 30 s, 72ºC for 1 min; and 1 cycle at 72ºC for 7 min. Products of the second round PCR were then recovered and gel-purified using the Universal DNA Purification Kit (Tiangen). Purified fragments were sub-cloned into a PMD18T-vector (Takara, Japan). Clones confirmed by PCR were selected for DNA sequencing. Snap Gene 2.3.2 software was used to blast the sequence.

### Statistical analysis

Data were presented as mean ± SEM. Statistical analyses were made using Prism 5 software (GraphPad, San Diego, CA, USA) with analysis of variance (ANOVA) where appropriate. The fluorescent intensity was calculated by NIH Image J software. *P* < 0.05 was significantly different.

## References

[1] Yanagimachi R, Chang MC. Fertilizable life of golden hamster ova and their morphological changes at the time of losing fertilizability. J Exp Zool. 1961; 148:185–203. 10.1002/jez.1401480303 14008938

[2] Miao YL, Kikuchi K, Sun QY, Schatten H. Oocyte aging: cellular and molecular changes, developmental potential and reversal possibility. Hum Reprod Update. 2009; 15:573–85. 10.1093/humupd/dmp014 19429634

[3] Lord T, Nixon B, Jones KT, Aitken RJ. Melatonin prevents postovulatory oocyte aging in the mouse and extends the window for optimal fertilization in vitro. Biol Reprod. 2013; 88:1–9. 10.1095/biolreprod.112.106450 23365415

[4] Dai X, Lu Y, Zhang M, Miao Y, Zhou C, Cui Z, Xiong B. Melatonin improves the fertilization ability of post-ovulatory aged mouse oocytes by stabilizing ovastacin and Juno to promote sperm binding and fusion. Hum Reprod. 2017; 32:598–606. 10.1093/humrep/dew362 28137755

[5] Gao YY, Chen L, Wang T, Nie ZW, Zhang X, Miao YL. Oocyte aging-induced Neuronatin (NNAT) hypermethylation affects oocyte quality by impairing glucose transport in porcine. Sci Rep. 2016; 6:36008. 10.1038/srep36008 27782163 PMC5080544

[6] Liang XW, Ge ZJ, Wei L, Guo L, Han ZM, Schatten H, Sun QY. The effects of postovulatory aging of mouse oocytes on methylation and expression of imprinted genes at mid-term gestation. Mol Hum Reprod. 2011; 17:562–67. 10.1093/molehr/gar018 21427161

[7] Tarín JJ, Pérez-Albalá S, Aguilar A, Miñarro J, Hermenegildo C, Cano A. Long-term effects of postovulatory aging of mouse oocytes on offspring: a two-generational study. Biol Reprod. 1999; 61:1347–55. 10.1095/biolreprod61.5.1347 10529284

[8] Tarín JJ, Pérez-Albalá S, Pérez-Hoyos S, Cano A. Postovulatory aging of oocytes decreases reproductive fitness and longevity of offspring. Biol Reprod. 2002; 66:495–99. 10.1095/biolreprod66.2.495 11804967

[9] Stehle JH, Saade A, Rawashdeh O, Ackermann K, Jilg A, Sebestény T, Maronde E. A survey of molecular details in the human pineal gland in the light of phylogeny, structure, function and chronobiological diseases. J Pineal Res. 2011; 51:17–43. 10.1111/j.1600-079X.2011.00856.x 21517957

[10] Liang S, Guo J, Choi JW, Kim NH, Cui XS. Effect and possible mechanisms of melatonin treatment on the quality and developmental potential of aged bovine oocytes. Reprod Fertil Dev. 2017; 29:1821–31. 10.1071/RD16223 27780517

[11] Wang T, Gao YY, Chen L, Nie ZW, Cheng W, Liu X, Schatten H, Zhang X, Miao YL. Melatonin prevents postovulatory oocyte aging and promotes subsequent embryonic development in the pig. Aging (Albany NY). 2017; 9:1552–64. 10.18632/aging.101252 28657543 PMC5509455

[12] Lord T, Aitken RJ. Oxidative stress and ageing of the post-ovulatory oocyte. Reproduction. 2013; 146:R217–27. 10.1530/REP-13-0111 23950493

[13] Miao Y, Zhou C, Cui Z, Zhang M, ShiYang X, Lu Y, Xiong B. Postovulatory aging causes the deterioration of porcine oocytes via induction of oxidative stress. FASEB J. 2018; 32:1328–37. 10.1096/fj.201700908R 29109171 PMC5892730

[14] Zhang T, Zhou Y, Li L, Wang HH, Ma XS, Qian WP, Shen W, Schatten H, Sun QY. SIRT1, 2, 3 protect mouse oocytes from postovulatory aging. Aging (Albany NY). 2016; 8:685–94. 10.18632/aging.100911 26974211 PMC4925822

[15] Tan DX, Manchester LC, Terron MP, Flores LJ, Reiter RJ. One molecule, many derivatives: a never-ending interaction of melatonin with reactive oxygen and nitrogen species? J Pineal Res. 2007; 42:28–42. 10.1111/j.1600-079X.2006.00407.x 17198536

[16] SanMiguel JM, Bartolomei MS. DNA methylation dynamics of genomic imprinting in mouse development. Biol Reprod. 2018; 99:252–62. 10.1093/biolre/ioy036 29462489 PMC6044325

[17] Uysal F, Akkoyunlu G, Ozturk S. Dynamic expression of DNA methyltransferases (DNMTs) in oocytes and early embryos. Biochimie. 2015; 116:103–13. 10.1016/j.biochi.2015.06.019 26143007

[18] Ma JY, Liang XW, Schatten H, Sun QY. Active DNA demethylation in mammalian preimplantation embryos: new insights and new perspectives. Mol Hum Reprod. 2012; 18:333–40. 10.1093/molehr/gas014 22447119

[19] Liang X, Ma J, Schatten H, Sun Q. Epigenetic changes associated with oocyte aging. Sci China Life Sci. 2012; 55:670–76. 10.1007/s11427-012-4354-3 22932882

[20] Ge ZJ, Schatten H, Zhang CL, Sun QY. Oocyte ageing and epigenetics. Reproduction. 2015; 149:R103–14. 10.1530/REP-14-0242 25391845 PMC4397590

[21] Wang H, Jo YJ, Oh JS, Kim NH. Quercetin delays postovulatory aging of mouse oocytes by regulating SIRT expression and MPF activity. Oncotarget. 2017; 8:38631–41. 10.18632/oncotarget.16219 28418847 PMC5503559

[22] Huang JC, Yan LY, Lei ZL, Miao YL, Shi LH, Yang JW, Wang Q, Ouyang YC, Sun QY, Chen DY. Changes in histone acetylation during postovulatory aging of mouse oocyte. Biol Reprod. 2007; 77:666–70. 10.1095/biolreprod.107.062703 17582009

[23] Liang XW, Zhu JQ, Miao YL, Liu JH, Wei L, Lu SS, Hou Y, Schatten H, Lu KH, Sun QY. Loss of methylation imprint of Snrpn in postovulatory aging mouse oocyte. Biochem Biophys Res Commun. 2008; 371:16–21. 10.1016/j.bbrc.2008.03.105 18381202

[24] Heinzmann J, Mattern F, Aldag P, Bernal-Ulloa SM, Schneider T, Haaf T, Niemann H. Extended in vitro maturation affects gene expression and DNA methylation in bovine oocytes. Mol Hum Reprod. 2015; 21:770–82. 10.1093/molehr/gav040 26155800

[25] Yang Q, Dai S, Luo X, Zhu J, Li F, Liu J, Yao G, Sun Y. Melatonin attenuates postovulatory oocyte dysfunction by regulating SIRT1 expression. Reproduction. 2018; 156:81–92. 10.1530/REP-18-0211 29752296

[26] Liang QX, Lin YH, Zhang CH, Sun HM, Zhou L, Schatten H, Sun QY, Qian WP. Resveratrol increases resistance of mouse oocytes to postovulatory aging *in vivo.* Aging (Albany NY). 2018; 10:1586–96. 10.18632/aging.101494 30036861 PMC6075442

[27] Qiao J, Chen Y, Yan LY, Yan J, Liu P, Sun QY. Changes in histone methylation during human oocyte maturation and IVF- or ICSI-derived embryo development. Fertil Steril. 2010; 93:1628–36. 10.1016/j.fertnstert.2009.03.002 19394606

[28] Yu C, Fan X, Sha QQ, Wang HH, Li BT, Dai XX, Shen L, Liu J, Wang L, Liu K, Tang F, Fan HY. CFP1 regulates histone H3K4 trimethylation and developmental potential in mouse oocytes. Cell Reports. 2017; 20:1161–72. 10.1016/j.celrep.2017.07.011 28768200

[29] Liu X, Wang C, Liu W, Li J, Li C, Kou X, Chen J, Zhao Y, Gao H, Wang H, Zhang Y, Gao Y, Gao S. Distinct features of H3K4me3 and H3K27me3 chromatin domains in pre-implantation embryos. Nature. 2016; 537:558–62. 10.1038/nature19362 27626379

[30] Han J, Wang QC, Zhu CC, Liu J, Zhang Y, Cui XS, Kim NH, Sun SC. Deoxynivalenol exposure induces autophagy/apoptosis and epigenetic modification changes during porcine oocyte maturation. Toxicol Appl Pharmacol. 2016; 300:70–76. 10.1016/j.taap.2016.03.006 26988607

[31] Zhang Y, Jia RX, Pan MH, Lu Y, Cui XS, Kim NH, Sun SC. HT-2 toxin affects development of porcine parthenotes by altering DNA and histone methylation in oocytes matured in vitro. Theriogenology. 2017; 103:110–16. 10.1016/j.theriogenology.2017.07.052 28780481

[32] Lan M, Han J, Pan MH, Wan X, Pan ZN, Sun SC. Melatonin protects against defects induced by deoxynivalenol during mouse oocyte maturation. J Pineal Res. 2018; 65:e12477. 10.1111/jpi.12477 29453798

[33] Lucifero D, Mertineit C, Clarke HJ, Bestor TH, Trasler JM. Methylation dynamics of imprinted genes in mouse germ cells. Genomics. 2002; 79:530–38. 10.1006/geno.2002.6732 11944985

[34] Stewart KR, Veselovska L, Kelsey G. Establishment and functions of DNA methylation in the germline. Epigenomics. 2016; 8:1399–413. 10.2217/epi-2016-0056 27659720 PMC5066131

[35] Han L, Ren C, Li L, Li X, Ge J, Wang H, Miao YL, Guo X, Moley KH, Shu W, Wang Q. Embryonic defects induced by maternal obesity in mice derive from Stella insufficiency in oocytes. Nat Genet. 2018; 50:432–42. 10.1038/s41588-018-0055-6 29459681

[36] Korkmaz A, Rosales-Corral S, Reiter RJ. Gene regulation by melatonin linked to epigenetic phenomena. Gene. 2012; 503:1–11. 10.1016/j.gene.2012.04.040 22569208

[37] Mackin SJ, O’Neill KM, Walsh CP. Comparison of DNMT1 inhibitors by methylome profiling identifies unique signature of 5-aza-2'deoxycytidine. Epigenomics. 2018; 10:1085–101. 10.2217/epi-2017-0171 30070602

[38] He B, Yin C, Gong Y, Liu J, Guo H, Zhao R. Melatonin-induced increase of lipid droplets accumulation and in vitro maturation in porcine oocytes is mediated by mitochondrial quiescence. J Cell Physiol. 2018; 233:302–12. 10.1002/jcp.25876 28240360

[39] Pasculli B, Barbano R, Parrella P. Epigenetics of breast cancer: biology and clinical implication in the era of precision medicine. Semin Cancer Biol. 2018; 51:22–35. 10.1016/j.semcancer.2018.01.007 29339244

[40] Zelic R, Fiano V, Grasso C, Zugna D, Pettersson A, Gillio-Tos A, Merletti F, Richiardi L. Global DNA hypomethylation in prostate cancer development and progression: a systematic review. Prostate Cancer Prostatic Dis. 2015; 18:1–12. 10.1038/pcan.2014.45 25384337

[41] Schwimmer H, Metzer A, Pilosof Y, Szyf M, Machnes ZM, Fares F, Harel O, Haim A. Light at night and melatonin have opposite effects on breast cancer tumors in mice assessed by growth rates and global DNA methylation. Chronobiol Int. 2014; 31:144–50. 10.3109/07420528.2013.842925 24131150

[42] Nie JY, Zhu XX, Xie BK, Nong SQ, Ma QY, Xu HY, Yang XG, Lu YQ, Lu KH, Liao YY, Lu SS. Successful cloning of an adult breeding boar from the novel Chinese Guike No. 1 swine specialized strain. 3 Biotech. 2016; 6:218. 10.1007/s13205-016-0525-4 28330290 PMC5055876

[43] Liang XW, Ge ZJ, Guo L, Luo SM, Han ZM, Schatten H, Sun QY. Effect of postovulatory oocyte aging on DNA methylation imprinting acquisition in offspring oocytes. Fertil Steril. 2011; 96:1479–84. 10.1016/j.fertnstert.2011.09.022 21982284

